# Spirituality in Professional Patient-Centered Care for Adults with Primary Brain Tumors: An Exploratory Scoping Review

**DOI:** 10.1007/s10943-024-02161-x

**Published:** 2024-11-05

**Authors:** Reinhard Grabenweger, Daniela Völz, Christiane Weck, Peter Hau, Piret Paal, Elisabeth Bumes

**Affiliations:** 1 Institute of Nursing Science and Practice, Paracelsus Medical University, Strubergasse 21, 5020 Salzburg, Austria; 2 Department of Neurology and Wilhelm Sander - NeuroOncology Unit, Regensburg University Hospital, Regensburg, Germany; 3 Institute of Palliative Care, Paracelsus Medical University, Salzburg, Austria; 4 Department of Ethnology, Institute of Cultural Studies, Tartu University, Tartu, Estonia

**Keywords:** Spirituality, Spiritual care, Brain neoplasms, Glioma, Palliative care

## Abstract

People with primary brain tumors face spiritual challenges due to neuro-cognitive disturbances such as aphasia and rapid neurological deterioration. This scoping review aimed to map the evidence regarding professional spiritual care in this neuro-oncological population. The literature search was conducted utilizing two databases (PubMed, CINAHL) and two peer-reviewed journals (‘Spiritual Care’, ‘Journal of Religion and Health’). In total, 27 studies were analyzed, and five thematic categories were identified: (1) spiritual needs, (2) integrating the spiritual dimension in care, (3) spiritual care generalist interventions, (4) spiritual care specialist interventions, (5) assessments. Early integration of spiritual care and specific training are important.

**Supplementary Information:**

The online version contains supplementary material available at 10.1007/s10943-024-02161-x.

## Introduction

Patients diagnosed with brain tumors are affected by cancer as well as a neurological disease, which leads to multidimensional suffering from patients' and caregivers’ perspectives (Kluger et al., 2023; Pasman & Koekkoek, 2023). Severe psychiatric and neurological symptoms like seizures, hemiparesis and aphasia are often the first symptoms and particularly prevalent (Bortolato et al., 2017; Choong & Vokes, 2008; Mukand et al., 2001; Mummudi & Jalali, 2014). Disease trajectories are characterized by neuro-cognitive disturbances and often rapid neurological deterioration, highlighting the need for early palliative care approaches including advance care planning (Fritz et al., 2022; Pace et al., 2017; Pasman & Koekkoek, 2023). Better integration of palliative care in neuro-oncological care, with reference to the availability of services and timing, is demanded (Pasman & Koekkoek, 2023). Loss of meaning, loss of identity, demoralization or hopelessness, loss of functional independence, prognostic uncertainty, and challenges to one’s faith are factors affecting the spiritual dimension of total pain in patients with neurological diseases (Kluger et al., 2023) and are described as antecedents of spiritual distress (Martins et al., 2024).

People with primary brain tumors, the population of interest in this article, differ from patients with other cancer entities in their disease trajectory and life expectancy which still remains limited (Pace et al., 2017, 2022). For example, patients with glioblastoma have a median overall survival of approximately 16 months (Stupp et al., 2017). A recent umbrella review revealed a lack of research on spirituality, religion and health among different oncological patient groups (Palmer Kelly et al., 2022). The authors emphasized the diversity of patient experiences in specific cancer diagnoses, which was one of the reasons for conducting this scoping review which focuses on patients with primary malignant brain tumors.

According to the European Association for Palliative Care (EAPC), spirituality is defined as the following:

“*Spirituality is the dynamic dimension of human life that relates to the way persons (individual and community) experience, express and/or seek meaning, purpose and transcendence, and the way they connect to the moment, to self, to others, to nature, to the significant and/or the sacred.*” (Nolan et al., 2011, p. 88)

In this paper, the adjective *spiritual* is used as a universal adjective, including the terms *existential* and *religious* (Best et al., 2020).

Spiritual care is valuable in considering the spiritual dimension in professional health care (Best et al., 2020, 2023). It is part of a holistic approach to patient-centered care and aims to improve the mental health, quality of life, and spiritual well-being of the patient and healthcare professional alike (Tavares et al., 2022). The following definition of spiritual care seems appropriate for Europe:

“*Spiritual care is that care which recognises and responds to the needs of the human spirit when faced with trauma, ill health or sadness and can include the need for meaning, for self-worth, to express oneself, for faith support, perhaps for rites or prayer or sacrament, or simply for a sensitive listener. Spiritual care begins with encouraging human contact in compassionate relationship, and moves in whatever direction need requires.*” (NHS Education for Scotland, 2009, p. 6)

To date, systematic reviews relevant to spiritual care practice in the field of neuro-oncology care have been published: two reviews on informal caregivers’ needs including spiritual needs (Applebaum et al., 2016; Sherwood et al., 2016), another review on symptom management and communication needs of patients with high-grade glioma (Crooms et al., 2022), and a systematic literature review revealing the underrepresentation of patients with primary brain tumors in the psycho-oncology literature (Loughan et al., 2021). Sprik and Tata (2021) provided a brief overview of religious/spiritual concerns of patients with brain cancer and their caregivers, but the authors did not present their review methods. The lack of a comprehensive and methodologically valid overview of spiritual care aspects in neuro-oncology prompted us to conduct this review following the scoping review methodology (Peters et al., 2020).

As evidence on spiritual care in patients with primary brain tumors provided by healthcare professionals is limited, our aim was to identify and present the available information regarding spiritual care in these patients within a professional healthcare setting. The primary research question was: What is the available evidence regarding professional spiritual care in patients with primary brain tumors? The sub-questions were: What specific spiritual needs are described within the sources of evidence identified in relation to the primary review question? Is there evidence of spiritual care interventions provided by health care professionals in this patient cohort? Is there information on other aspects relevant to spiritual care for patients with primary brain tumors?

## Method

**Fig. 1:**
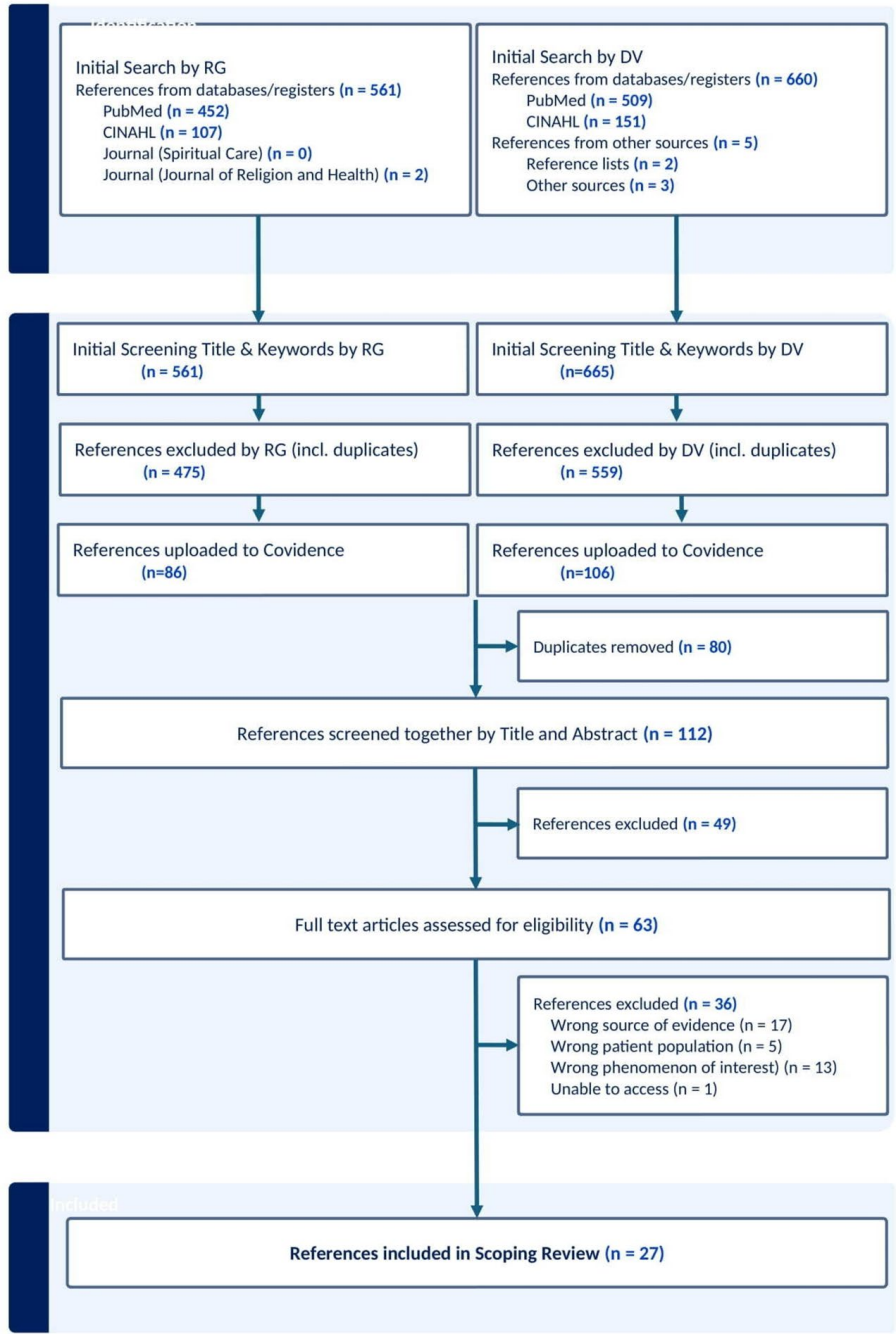
PRISMA Flowchart:Selection Process

We conducted this scoping review with respect to the Joanna Briggs Institute’s (JBI) updated guidance for the conduct of scoping reviews (Peters et al., 2020). The scoping review design is suitable for mapping evidence in the field of spiritual care for people with primary malignant brain tumors. Due to the initial independent works of the first authors RG and DV, we had to adapt some points of the recommended way of conducting a scoping review. This scoping review was not registered because of the dynamic and iterative research process. RG has already conducted an initial literature search for his master’s thesis before the decision to collaborate with DV was made to provide room for a deeper and more coherent analysis of the available resources. The grown character of the review is also reflected in the search strategy, which was not developed with the help of a scientific librarian. The presented flowchart enhances the transparency of the altered literature search and screening process (Fig. 1).

### Eligibility Criteria

The authors agreed upon the following inclusion criteria before conducting the literature search (Table 1). According to the JBI guidance for scoping reviews (Aromataris et al., 2024; Peters et al., 2020) and the defined research questions, all quality evidence was included in this scoping review, and no limits concerning the date of publication were set. Other forms of evidence syntheses that met the inclusion criteria were reviewed for additional evidence. Other than recommended by JBI we did not define context criteria, as this seemed irrelevant to answer the research questions.

**Table 1:** Eligibility Criteria

Element	Inclusion criteria	Exclusion criteria
Population	Adult patients with a primary malignant brain tumor	Patients under the age of 18 and patients with other central nervous system tumors
Concept	Spiritual care^NHS Education for Scotland. (2009)^ (spiritual needs, interventions, outcomes, other relevant aspects)	Other conceptualizations of spiritual care
Types of evidence sources	RCTs; qualitative studies; quantitative studies; mixed-methods studies; pro- and retrospective studies; case studies; case–control studies; abstracts; dissertations; editorials	evidence syntheses (systematic and scoping reviews, meta-analyses, meta-syntheses); webpages; books; blogs

### Search Strategy

Initially, the author RG conducted a search of the databases PubMed and CINAHL (Cumulative Index to Nursing and Allied Health Literature) to identify relevant articles, keywords, and index terms. Subsequently, the authors RG and DV carried out an electronic search independently in the CINAHL and PubMed databases with the last search on 27th of February 2023. Each reviewer (RG and DV) used different search strategies and keywords to ensure a comprehensive and broad representation of the available literature. Boolean operators were used to combine search terms. RG also used Medical Subject Headings (MeSH) and CINAHL Subject Headings when searching the corresponding database. The complete search strategy can be found in Online Appendix 1. Moreover, identified reviews were analyzed, and their reference lists were used as additional resources. Additionally, the journals Spiritual Care and Journal of Religion and Health were screened for relevant publications (RG).

### Evidence Screening and Selection

Initially, every search result was screened by reading the titles and keywords directly in PubMed and CINAHL (Fig. 1). The citations of all papers that fit the inclusion criteria were uploaded into the Covidence software and duplicates were removed. Subsequently, each of the two reviewers (RG and DV) independently screened the abstracts for the eligibility criteria. In case of conflicts, a third reviewer (EB) decided whether an article was included or excluded. Full-text screening was performed on the remaining articles. Any queries concerning the reason for exclusion were discussed by both reviewers (RG and DV), and a final list of studies was created.

### Data Extraction

The data were extracted based on the research questions and objectives of this scoping review. The authors agreed on a self-developed data extraction sheet following the JBI Manual recommendations for evidence charting for scoping reviews (Aromataris et al., 2024). The following data items were presented on an Excel spreadsheet (Tabel 2): title, authors, year of publication, study design, sample size and population, participants' race/ethnicity and religious affiliation, country and setting, aim/purpose of the study, key findings regarding professional spiritual care, and implications for healthcare workers. Data extraction was independently performed by the reviewers (RG and DV). The results were then compared, and possible conflicts were resolved. The full worksheets are shown in Online Appendix 2 and were used for the synthesis of the results.

### Data Analysis and Synthesis of Results

Data analysis was done by RG and DV. After initial immersion in the data through reading, a basic coding approach was used (Pollock et al., 2023). Due to the broad scope of this scoping review and poor evidence on our research topic, the authors chose an inductive approach to develop a coding framework and perform data categorization. In addition, some codes were developed deductively by referring to the spiritual care generalist and specialist model of multi-disciplinary spiritual care provision in palliative care (Best et al., 2020). The data were synthesized into the final findings by RG and discussed by all the authors to reach a consensus. The results are presented in descriptive and tabular forms in the following section.

## Results

In this scoping review, 27 publications were included with the aim of mapping the evidence of professionals’ provision of spiritual care for patients with primary brain tumors (Fig. 1). The flow chart presented in Fig. 1 demonstrates the process of data selection. Online Appendix 2 summarizes the data extraction of the publications included in this scoping review. The results are grouped in characteristics of sources of evidence and themes.

### Characteristics of Sources of Evidence

The majority of the studies included in this scoping review were qualitative (n = 11) (Adelbratt & Strang, 2000; Cavers et al., 2012; Korman et al., 2021; Lipsman et al., 2007; Loughan et al., 2022a; Nixon & Narayanasamy, 2010; Nixon et al., 2013; Philip et al., 2014; Piderman et al., 2017a; Ravishankar & Bernstein, 2014; Strang et al., 2001). There were four case studies (Brody et al., 2004; Khalili, 2007; Roberts & Applebaum, 2022; Vedelø et al., 2018), three retrospective analyses (Hyer et al., 2021; Kuchinad et al., 2017; Randazzo et al., 2021), three mixed-methods studies (Mehta et al., 2018; Philip et al., 2020; Piderman et al., 2015b), two quantitative studies (Piderman et al., 2015a, 2017b), and one randomized-controlled trial study (Ownsworth et al., 2015), as well as one proof-of-concept trial study (Loughan et al., 2022b) included. In addition, two opinion papers (Elia et al., 2020; Sprik et al., 2021) were considered in the analysis. The included studies were published between 2000 and 2022.

**Fig. 2:**
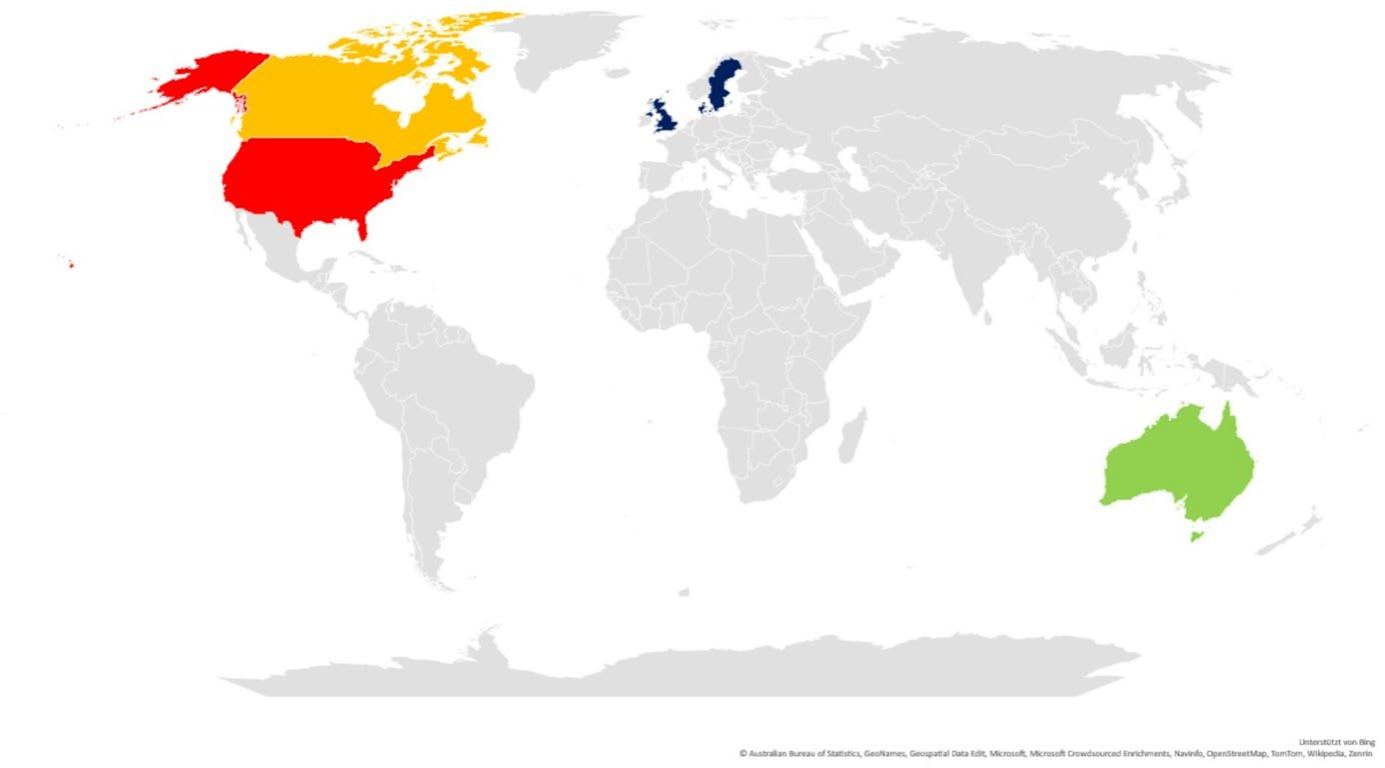
Spatial Distribution and Cultural Background

Concerning the cultural background, 14 of the included publications were from the USA (Brody et al., 2004; Elia et al., 2020; Hyer et al., 2021; Kuchinad et al., 2017; Loughan et al., 2022a, 2022b; Mehta et al., 2018; Piderman et al., 2015a, b, 2017a, b; Randazzo et al., 2021; Roberts & Applebaum, 2022; Sprik et al., 2021). Four Canadian studies (Khalili, 2007; Korman et al., 2021; Lipsman et al., 2007; Ravishankar & Bernstein, 2014) and three Australian studies (Ownsworth et al., 2015; Philip et al., 2014, 2020) were included. Six studies were conducted in Europe: three in the United Kingdom (Cavers et al., 2012; Nixon & Narayanasamy, 2010; Nixon et al., 2013), two in Sweden (Adelbratt & Strang, 2000; Strang et al., 2001), and one in Denmark (Vedelø et al., 2018) (Fig. 2).

Most studies did not report cultural or ethnic diversity in the population of interest. Two studies reported a maximum variation sampling strategy considering the participants’ ethnicity or ethnic origin (Adelbratt & Strang, 2000; Strang et al., 2001), but provided no detailed information on the participants’ ethnicity. The authors of five articles provided information on race (Hyer et al., 2021; Kuchinad et al., 2017; Loughan et al., 2022a, b; Roberts & Applebaum, 2022), however, the majority of the populations under study were white Caucasians. Eight studies considered religious affiliations (Hyer et al., 2021; Lipsman et al., 2007; Piderman et al., 2015a, b, 2017a, b; Randazzo et al., 2021; Ravishankar & Bernstein, 2014).

Despite the broad scope of this review, participants' characteristics and study contexts are not diverse, as all studies are conducted in countries with high incomes and reasonably good palliative care services. Among the study participants, almost all studies included patients with different tumor grades and in multiple stages of the disease. In five studies, patients with brain tumors and their informal caregivers were investigated (Adelbratt & Strang, 2000; Lipsman et al., 2007; Philip et al., 2020; Piderman et al., 2017b; Roberts & Applebaum, 2022). Additional four publications included patients, their next of kin and healthcare professionals (Cavers et al., 2012; Khalili, 2007; Korman et al., 2021; Strang et al., 2001). One study involved only nurses (Nixon et al., 2013) and another study included only colleagues from neuro-oncology fellowship programs (Mehta et al., 2018).

### Themes

**Fig. 3:**
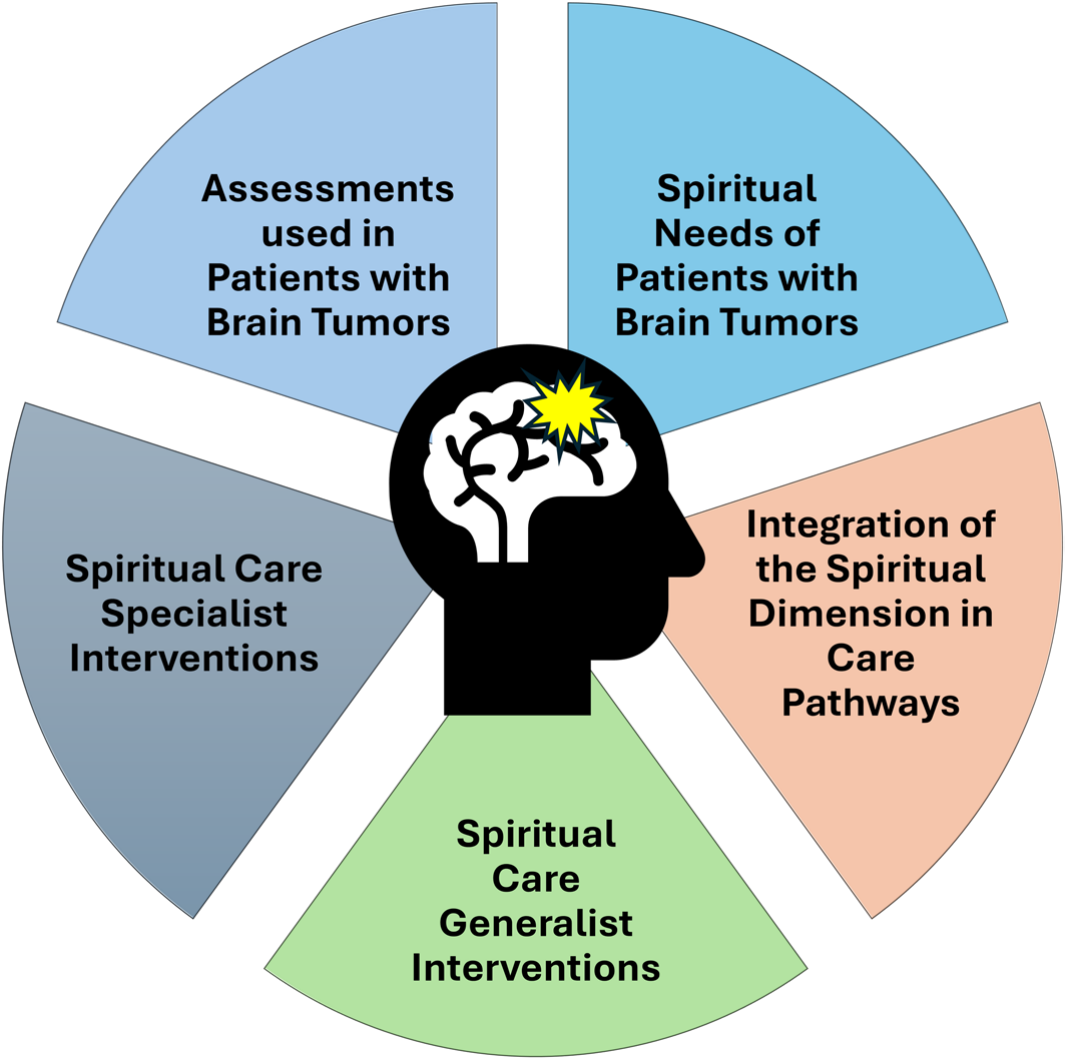
Identified Themes

Within the selected publications, five themes were generated: (1) spiritual needs of patients with brain tumors, (2) integration of the spiritual dimension in care pathways, (3) spiritual care generalist interventions, (4) spiritual care specialist interventions, and (5) assessments used in patients with primary brain tumors (Fig. 3). The themes are presented in narrative form and additional information is provided, when appropriate, in tables.

### Spiritual Needs of Patients with Brain Tumors

The included studies showed that patients with brain tumors had varying spiritual needs. These needs can change during the disease trajectory, highlighting the dynamic dimension of spirituality (Nolan et al., 2011; Philip et al., 2020). Philip et al. (2020) demonstrated that spiritual needs were more acute at initial diagnosis, subsequently decreased with hospital therapy, and increased again during outpatient therapy, which makes consistent screening for these changing levels of spiritual distress essential. However, the spiritual needs of patients on a neurosurgical unit are not always adequately met by nurses (Nixon & Narayanasamy, 2010; Nixon et al., 2013). In an Australian study focusing on the disease trajectory, the authors described that patients felt that existential issues were neglected compared to medical issues (Philip et al., 2014). Thus, discussing existential issues should be an integral part of advance care planning.

Furthermore, patients with brain tumors have a need for existential support with a focus on interpersonal relationships and open communication (Adelbratt & Strang, 2000; Cavers et al., 2012; Lipsman et al., 2007). Due to individual information and communication needs of vulnerable patients, communication must be adapted based on trustworthy relationships with healthcare professionals (Vedelø et al., 2018). A retrospective, cross-sectional study from the U.S. highlighted that spiritual well-being is important for enhancing the health-related quality of life (HRQoL) (Randazzo et al., 2021). The authors recommend assessments of spiritual needs at every clinical visit to cater to the patient’s changing needs after a brain tumor diagnosis.

The following spiritual needs were identified in a qualitative study from the patients’ perspective: “*supportive family relationships, emotional support, loneliness, religious needs, need to talk, reassurance, anxiety, solitude, denial, plans for the future, thoughts about meaning of life, end of life decisions, […] discussion of beliefs*” (Nixon & Narayanasamy, 2010, p. 2259). Out of the neurosurgical nurses’ perspectives the “*need to talk about spiritual concerns, showing sensitivity to patients’ emotions, [and] responding to religious needs*” (Nixon et al., 2013, p. 1) are recognized. Nixon et al. (2013) also report relatives’ spiritual needs (“*supporting them with end of life decisions, supporting them when feeling being lost and unbalanced, encouraging exploration of meaning of life, and providing space, time and privacy to talk*” (Nixon et al., 2013, p. 1)).

### Integrating the Spiritual Dimension in Care Pathways

Although studies have shown that patients with malignant brain tumors have particular spiritual needs, the integration of the spiritual dimension in care pathways is not always successful. Spiritual matters may not be documented in practice (Kuchinad et al., 2017) and healthcare professionals may feel uncomfortable when they have to address spiritual distress in their patients (Mehta et al., 2018). Similar findings were reported when asking British nurses, even though they reported theoretical knowledge on the acknowledgement of spiritual needs (Nixon et al., 2013). A US-American retrospective study indicates that individuals with bad prognosis tumors like brain tumors had greater odds of utilizing pastoral care than other cancer entities (Hyer et al., 2021).

The individuals’ disease trajectories must be considered when it comes to the integration of spiritual care. In this context, spirituality is described as a possible coping mechanism and recommendations for acknowledging the existential concerns of patients are made to improve patient-centered care in neuro-oncology (Loughan et al., 2022a). Multiple changes and transitions are experienced by patients during the disease trajectory and were explicitly thematized. These transitions include uncertainty around the initial diagnosis, followed by coping with the life-changing diagnosis or the beginning of neurocognitive decline, which then requires more intense care and results in a loss of autonomy (Brody et al., 2004; Khalili, 2007; Ravishankar & Bernstein, 2014).

A multi-professional team approach may be valuable for addressing the spiritual dimension of care (Brody et al., 2004). Khalili (2007) emphasized the important role of nurses. Nurses should know strategies like *“active listening, highlighting strengths, promoting hope and providing and clarifying information”* (Khalili, 2007, p. 12) and use them repeatedly. Concerning the active treatment stage—especially when it comes to surgery—Ravishankar and Bernstein (2014) studied the role of religion influencing coping mechanisms in the time around craniotomy. They concluded that patients prefer to have an explicit time and space for their religious practices and gain spiritual strength through them, but do not want their physicians to join (Ravishankar & Bernstein, 2014).

Facing the end of life, Elia et al. (2020) point out in their opinion paper about the end-of-life care of patients with meningioma that a *transdisciplinary team* model including chaplains would be necessary to improve palliative care, which has also been suggested by Mehta et al. (2018). The authors also represent the opinion that physicians and nurses should be able to conduct basic assessments of psychosocial and spiritual needs, and recommend specialized spiritual care for patients with speech impairments (Elia et al., 2020).

Philip et al. (2014) found that patients with primary malignant brain tumors have complex existential and psychosocial needs. However, they described an existing *“gap in services”* (Philip et al., 2014, p. 389) to address these needs. They highlighted that the focus should be on immediate needs because of the pervasive loss of the patients’ former sense of self.

### Spiritual Care Interventions for Spiritual Care Generalists

Analysis of studies including healthcare team members such as doctors and nurses providing spiritual care shows that these professionals know some strategies to meet the spiritual needs of neuro-oncological patients. British patients reported following nurses’ strategies to address their spiritual needs: being present, “*being flexible with hospital policies, encouraging family relationships, providing privacy and providing religious support*” (Nixon & Narayanasamy, 2010, p. 2267). Similar behaviors have been reported by neurosurgical nurses (Nixon et al., 2013). However, the nurses were not always aware of existing spiritual needs, partially because of the acute nature of the situation.

Open communication and support of general practitioners are strategies to enhance the well-being of patients with glioma (Cavers et al., 2012). These strategies are especially important during the initial stages of illness at the time of diagnosis when spiritual needs are more prevalent (Philip et al., 2020). According to the case study of Khalili (2007), which thematizes the ongoing transitions of a patient with glioblastoma, nurses are very important in assessing a patient’s need to discuss end-of-life issues. However, some nurses are unsure what strategies are meant by existential support and many of them do not see this kind of support as their duty (Strang et al., 2001). The team approach is also addressed by Brody et al. (2004) who demonstrated that a spiritual counselor could be an important member of the caring team of a patient with brain tumor.

### Spiritual Care Interventions for Spiritual Care Specialists

In this scoping review we have identified some studies on specialized spiritual care interventions based on the complex spiritual and existential needs of brain tumor patients. These interventions were developed and tested for spiritual care specialists, such as chaplains and psychotherapists, as well as trained healthcare professionals. Online Appendix 3 presents the interventions, their description, the study populations/participants, the outcomes, and the assessments used.

The following spiritual care interventions were identified in this scoping review:

- *Dignity Therapy (DT)* (Korman et al., 2021)
- *Managing Cancer and Living Meaningfully (CALM*) (Loughan et al., 2022b)
- *Making Sense of Brain Tumor (MSoBT)* (Ownsworth et al., 2015)
- *Hear my Voice* (Piderman et al., 2015a, b, 2017a, b; Sprik et al., 2021)
- *Meaning-Centered Psychotherapy (MCP) & Meaning-Centered Psychotherapy for Caregivers (MCP-C)* (Roberts & Applebaum, 2022)

Generally, the interventions were designed to support patients and their informal caregivers at the end of their life. Differences in treatment lengths were noticed, ranging from programs with two sessions in one to two weeks (Korman et al., 2021) to weekly one-hour sessions for seven or ten weeks (Ownsworth et al., 2015; Roberts & Applebaum, 2022).

Involvement of informal caregivers and family also varies: The *Making Sense of Brain Tumor* (Ownsworth et al., 2015) and the *Meaning-Centered Psychotherapy & Meaning-Centered Psychotherapy for Caregivers* (Roberts & Applebaum, 2022) acknowledge the importance of the caregivers. The *Hear my Voice* (Piderman et al., 2017b) program focuses on patients telling their own spiritual history, but their support persons may join as well.

*Dignity Therapy* can be offered to patients by trained members of the interprofessional health care team (Korman et al., 2021). The *Managing Cancer and Living Meaningfully (CALM)* (Loughan et al., 2022b), *Making Sense of Brain Tumor (MSoBT)* (Ownsworth et al., 2015) and *Meaning-Centered Psychotherapy (MCP) & Meaning-Centered Psychotherapy for Caregivers (MCP-C)* (Roberts & Applebaum, 2022) are psychotherapeutic programs. The *Hear my Voice* program is described as a program led by chaplains (Piderman et al., 2015a, b, 2017a, b; Sprik et al., 2021). This chaplain-led intervention includes a spiritual life review and proofed as a feasible intervention for people with brain cancer and their support persons.

### Assessments Used in Patients with Primary Brain Tumors

The assessments used in the included studies were heterogenous (Online Appendix 4). Notably, the *Functional Assessment of Chronic Illness Therapy (FACIT)* and *Functional Assessment of Cancer Therapy (FACT)* scales were used more often in this patient cohort than other assessments. Ownsworth et al. (2015) reflected on the validity of these assessments in patients with brain tumors and criticized the length of the FACT, which made them drop it from follow-up assessments in their study. In addition, Philip et al. (2020) report that the *Distress Thermometer* is a valid tool for the use in patients with brain tumors. This single-item instrument is seen as more favorable for use in brain tumor patients compared to longer instruments (Kvale et al., 2009). In the other papers, the reason for selecting these assessment tools for patients with brain tumors was not further explained.

It is important to know that some of the listed scales in Online Appendix 4 have been identified as contaminated scales because of an inadequate choice of items and tautologies. Those scales need to be used with great caution and the studies’ findings might be of limited evidence (Koenig, 2011; Koenig & Carey, 2024). Furthermore, the authors of this scoping review did not check content validity of the identified scales. However, as another exploratory review showed, some of the identified scales might not measure what is intended (Drummond & Carey, 2019).

## Discussion

### Main Findings

This scoping review aimed to identify and present available information regarding spiritual care in patients with primary brain tumors provided in a professional healthcare context. Our data synthesis revealed that healthcare professionals do not always meet their spiritual needs adequately, which may lead to the promotion of spiritual distress (Martins et al., 2024). Screening brain cancer patients for existential distress has been suggested, but the analysis of the assessments used in the included studies showed that neither validated instruments for assessing spiritual needs in clinical practice nor adequate outcome assessments for spiritual care interventions exist (Loughan et al., 2022a). We identified a lack of evidence on the topic of spiritual care in neuro-oncology and could not provide clear recommendations for clinical practice. More research is needed to enable evidence-based spiritual care in neuro-oncology.

In addition, the neurocognitive decline experienced by patients presents a challenge in researching aspects of spiritual care. The exclusion of participants with language and cognitive impairments leads to a selection bias (Adelbratt & Strang, 2000; Cavers et al., 2012; Lipsman et al., 2007; Philip et al., 2014; Piderman et al., 2017b). Moreover, there was a significant loss to follow-up due to death, and mental and physical deterioration (Ownsworth et al., 2015). This selection bias, as well as the difficulty in collecting qualitative data from patients with aphasia caused by their brain tumor was also discussed by Watanabe (2005). Whenever patients with brain tumors experience aphasia or neurocognitive impairments, the role of their informal caregivers can be essential for spiritual care provision (Applebaum et al., 2016; Sherwood et al., 2016).

To provide evidence-based spiritual care to patients with brain tumors, patients’ needs should be more explicitly studied through their eyes and not solely reported by their caregivers. In future studies, ways of dealing with expected neurocognitive decline need to be found to enable direct and longer research participation of patients. The suitability of assessments and interventions in brain tumor patients experiencing cognitive decline could be tested in a comparative setting with non-brain tumor patients.

Analysis of the included references showed a lack of reporting on cultural and ethnic diversity, which can be seen as an important aspect of professional culture-sensitive spiritual care. The cultural backgrounds of the included studies were not diverse, either, which might mirror international inequalities in the care of patients with brain tumors and the provision of palliative care. There is a general bias in spiritual care research in patients with brain tumors, as most studies come from Western countries with secular societies (Fig. 2).

A striking limitation of the current literature is the homogeneity of the patient population. While interventions like *Hear My Voice* are designed for spiritual specialists and led by chaplains, they may not fully address the diverse spiritual needs of patients. Although 5% of participants in the study identified as Muslim, the authors do not discuss the potential impact of religious differences (Piderman et al., 2017a).

Feasible concepts for spiritual care provision in changing health care environments have to be discussed. Strang et al. (2001) present obstacles to the provision of spiritual support in their qualitative study about existential support in patients with brain tumor and their spouses. Nurses, patients, and relatives predominantly report obstacles, such as lack of time and knowledge. Nixon et al. (2013) also describe nurses' inability to recognize situations in which patients may need spiritual care and their ability to reflect retrospectively on the subtle expression of patients' spiritual struggle, possibly due to lack of time in acute hospitals. This further highlights the need for structured education and training in the field of spiritual care for nurses, and the integration of a designated time for spiritual discourse in the care plan.

Owing to the broad scope of this scoping review, it must be noted that many publications address existential distress, which was recently identified as a surrogate term of spiritual distress by Martins et al. (2024). However, when it comes to nurses, existential distress is currently not listed as a separate NANDA-I nursing diagnosis, but only the Nursing Diagnosis of Spiritual Distress (Diagnosis Code 00066) (Herdman, 2021). Each publication was studied to prove the extent to which the findings regarding existential distress are relevant in the field of spiritual care. Altogether, the concept of existential distress seems to comprise multiple related themes as one possible taxonomy of existential concerns shows (Philipp et al., 2021). The proposed research model of existential distress in life-threatening illnesses such as advanced cancer seems applicable to this scoping review’s findings (Philipp et al., 2021). Furthermore, the process model of spiritual distress described by Martins et al. (2024) with identified antecedents, attributes, and outcomes is seen as applicable to this study’s findings. However, there is a current lack of studies explicitly addressing spiritual care and its interventions in multiprofessional team approaches, even though the WHO clearly includes the spiritual dimension in their palliative care guideline (World Health Organization, 2020).

### Strengths and Limitations

Although the authors of this scoping review acknowledged the JBI guidance for scoping reviews (Peters et al., 2020), they have to declare some limitations. First, the scoping review was not registered, and there was no published review protocol, but the authors agreed in advance on eligibility criteria and the search strategy. The authors decided to search solely in the databases PubMed and CINAHL for references, which must be seen as limitations in regard to breadth and comprehensiveness of the search. A scientific librarian was not consulted due to limited resources to conduct this scoping review.

Although the authors aimed to include all relevant publications available, irrespective of the publication language, the search with English search terms possibly excluded publications with titles and abstracts in another language. The selection of resources and data charting were primarily done independently by RG and DV, and the synthesis of results following a basic coding approach was mainly done by RG—a novice researcher in spiritual care with experiences in neurosurgery—and checked by DV and the other reviewers. All steps of the scoping review methodology process (Peters et al., 2020) were discussed and consensus among the authors was achieved, which can be seen as a strength.

In general, many included studies lack a precise definition of key terms like spiritual care or existential distress (Brody et al., 2004; Elia et al., 2020; Khalili, 2007; Kuchinad et al., 2017; Loughan et al., 2022b). When core concepts are not defined, the comparability of the results remains limited, and the concise inclusion and exclusion of individual studies is challenging, possibly leading to inconsistencies in data selection. RG and DV had detailed discussions with the other authors to reduce the risk of incoherence and carefully decided on the data selection. This complex and careful selection of sources of evidence is a further strength of this scoping review. Additionally, the broad scope of this scoping review allowed to map the existing evidence including a large variety of quality of evidence.

## Conclusions

This scoping review revealed that there is a clear need for spiritual care in patients with primary malignant brain tumors in the context of professional healthcare. The existing evidence emphasizes the importance of early palliative care approaches including spiritual care in neuro-oncological patients (Kluger et al., 2023; Pace et al., 2017, 2022). Besides, clinicians should know strategies how to encounter patients experiencing spiritual distress at different key points in the disease trajectory (Crooms et al., 2022). Further research is necessary to make spiritual care accessible to people, irrespective of their cultural background or ethnicity. Spiritual care implementation and evaluation studies are desirable, where the authors clearly state their spiritual care understanding and develop culture-sensitive and validated assessment instruments for spiritual distress in patients with primary malignant brain tumors. As there are currently few studies that give a voice to patients, and not merely to informal caregivers, new models to assess spiritual needs despite the beginning of cognitive deterioration and aphasia would provide better insights into patient-specific needs. These data could help guide the development and validation of specific interventions for spiritual care generalists in neuro-oncology and increase the rates of spiritual care provision for the thus far neglected brain tumor patients.

## Supplementary Information

Below is the link to the electronic supplementary material.

Supplementary file1 (DOCX 16 kb)

Supplementary file2 (XLSX 22 kb)

Supplementary file3 (XLSX 18 kb)

Supplementary file4 (DOCX 26 kb)
