# Supplementary material for: Spirituality in Professional Patient-Centered Care for Adults with Primary Brain Tumors: An Exploratory Scoping Review
Source: J Relig Health. 2024 Nov 5;64(3):2165–83. doi: 10.1007/s10943-024-02161-x (PMC12133965; doi:10.1007/s10943-024-02161-x)
Supplement: Supplementary file 1 — Supplementary file1 (DOCX 16 kb) [file 10943_2024_2161_MOESM1_ESM.docx]

Table 1: Search Strategy PubMed (MEDLINE)

| #1 | ("Religion and Psychology"[Mesh]) AND "Glioma"[Mesh] |
| --- | --- |
| #2 | ("Religion and Psychology"[Mesh]) AND "Brain Neoplasms"[Mesh] |
| #3 | (spiritual* OR religio* OR pastoral* OR existential*) AND neuro* AND oncolog* |
| #4 | (spiritual* OR religio* OR pastoral* OR existential*) AND gliom* |
| #5 | (spiritual* OR religio* OR pastoral* OR existential*) AND (brain* OR cerebral*) AND (cancer* OR tumo?r OR neoplasm*) |
| #6 | (Glioblastoma) AND (Spiritual) |
| #7 | (Glioblastoma) AND (Religious) |
| #8 | (Glioblastoma) AND (Existential) |
| #9 | (Glioma) AND (Spiritual) |
| #10 | (Glioma) AND (Religious) |
| #11 | (Glioma) AND (Existential) |
| #12 | (Ependymoma) AND (Spiritual) |
| #13 | (Ependymoma) AND (Religious) |
| #14 | (Ependymoma) AND (Existential) |
| #15 | (Oligodendroglioma) AND (Spiritual) |
| #16 | (Oligodendroglioma) AND (Religious) |
| #17 | (Oligodendroglioma) AND (Existential) |
| #18 | (Brain Tumor) AND (Spiritual) |
| #19 | (Brain Tumor) AND (Religious) |
| #20 | (Brain Tumor) AND (Existential) |
| #21 | (Brain Neoplasm) AND (Spiritual) |
| #22 | (Brain Neoplasm) AND (Religious) |
| #23 | (Brain Neoplasm) AND (Existential) |
| #24 | (Brain Cancer) AND (Spiritual) |
| #25 | (Brain Cancer) AND (Religious) |
| #26 | (Brain Cancer) AND (Existential) |
| #27 | (Astrocytoma) AND (Spiritual) |
| #28 | (Astrocytoma) AND (Religious) |
| #29 | (Astrocytoma) AND (Existential) |

Table 2: Search Strategy CINAHL (EBSCO)

| #1 | TI ( spiritual* OR religio* OR pastoral* OR existential* ) AND TI neuro* AND oncolog* |
| --- | --- |
| #2 | ( (spiritual* OR religio* OR pastoral* OR existential*) ) AND TI gliom* |
| #3 | ( (spiritual* OR religio* OR pastoral* OR existential*) ) AND TI ( (brain* OR cerebral*) ) AND TI ( (cancer* OR tumo?r OR neoplasm*) ) |
| #4 | ( (MH "Spiritual Care") OR (MH "Spirituality") ) AND (MH "Brain Neoplasms") |
| #5 | ( (MH "Spirituality") OR (MH "Religion and Religions") ) AND (MH "Brain Neoplasms") |
| #6 | (Glioblastoma) AND (Spiritual) |
| #7 | (Glioblastoma) AND (Religious) |
| #8 | (Glioblastoma) AND (Existential) |
| #9 | (Glioma) AND (Spiritual) |
| #10 | (Glioma) AND (Religious) |
| #11 | (Glioma) AND (Existential) |
| #12 | (Ependymoma) AND (Spiritual) |
| #13 | (Ependymoma) AND (Religious) |
| #14 | (Ependymoma) AND (Existential) |
| #15 | (Oligodendroglioma) AND (Spiritual) |
| #16 | (Oligodendroglioma) AND (Religious) |
| #17 | (Oligodendroglioma) AND (Existential) |
| #18 | (Brain Tumor) AND (Spiritual) |
| #19 | (Brain Tumor) AND (Religious) |
| #20 | (Brain Tumor) AND (Existential) |
| #21 | (Brain Neoplasm) AND (Spiritual) |
| #22 | (Brain Neoplasm) AND (Religious) |
| #23 | (Brain Neoplasm) AND (Existential) |
| #24 | (Brain Cancer) AND (Spiritual) |
| #25 | (Brain Cancer) AND (Religious) |
| #26 | (Brain Cancer) AND (Existential) |
| #27 | (Astrocytoma) AND (Spiritual) |
| #28 | (Astrocytoma) AND (Religious) |
| #29 | (Astrocytoma) AND (Existential) |
