# Supplementary material for: Spirituality in Professional Patient-Centered Care for Adults with Primary Brain Tumors: An Exploratory Scoping Review
Source: J Relig Health. 2024 Nov 5;64(3):2165–83. doi: 10.1007/s10943-024-02161-x (PMC12133965; doi:10.1007/s10943-024-02161-x)
Supplement: Supplementary file 4 — Supplementary file4 (DOCX 26 kb) [file 10943_2024_2161_MOESM4_ESM.docx]

| Assessments related to spirituality | (1) | (2) | (3) | (4) | (5) | (6) |
| --- | --- | --- | --- | --- | --- | --- |
| Patient Health Questionnaire (PHQ-9) | x |  |  |  |  |  |
| Death and Dying Distress Scale (DADDS) | x |  |  |  |  |  |
| Generalized Anxiety Disorder (GAD-7) | x |  |  |  |  |  |
| Quality of Life at the End-of-Life Cancer Scale (QUAL-EC) | x |  |  |  |  |  |
| Functional Assessment of Chronic Illness Therapy – Spiritual Wellbeing Scale (FACIT-sp) | x |  |  |  |  |  |
| Fear of Cancer Recurrence (FCR-7) | x |  |  |  |  |  |
| Clinical Evaluation Questionnaire (CEQ) | x |  |  |  |  |  |
| neuropsychological measures: Digit Span, Hopkins Verbal Learning Test, Rey Complex Figure, Trail Making Test and Verbal Fluency, global neuropsychological functioning (GNF) |  | x |  |  |  |  |
| McGill Quality of Life Questionnaire (MQOL) |  | x |  |  |  |  |
| McGill Quality of Life Questionnaire (MQOL-EW) (existential subscale) |  | x |  |  |  |  |
| Montgomery–Asberg Depression Rating Scale (MADRS) |  | x |  |  |  |  |
| Depression Anxiety Stress Scales—21 (DASS-21) |  | x |  |  |  |  |
| Functional Assessment of Cancer Therapy-Brain (FACT-Br) |  | x |  |  |  | x |
| Functional Assessment of Cancer Therapy-General (FACT-G) |  | x |  |  |  | x |
| Distress Thermometer |  |  | x |  |  |  |
| Functional Assessment of Chronic Illness Therapy-Spiritual Well-Being 12 (FACIT-Sp-12) |  |  |  | x |  | x |
| Brief Religious Coping Scale (Brief RCOPE) |  |  |  | x | x |  |
| Linear Analog Self-Assessment (LASA) |  |  |  | x | x |  |
| Functional Assessment of Chronic Illness Therapy-Spiritual Expanded Version (FACIT-Sp-Ex) |  |  |  |  | x |  |
| Linear Analog Self-Assessment (one item: How would you describe your spiritual well being?) |  |  |  |  | x |  |
| Brief COPE Inventory (COPE) (religion subscale) |  |  |  |  | x |  |
| Functional Assessment of Chronic Illness Therapy Fatigue (FACIT-F) |  |  |  |  |  | x |
| International Complementary and Alternative Medicine Questionnaire (i-CAM-Q) (modified) |  |  |  |  |  | x |

1. Loughan AR, Willis KD, Braun SE, Rodin G, Lanoye A, Davies AE, et al. Managing cancer and living meaningfully (CALM) in adults with malignant glioma: a proof-of-concept phase IIa trial. Journal of Neuro-Oncology. 2022;157(3):447-56.

2. Ownsworth T, Chambers S, Damborg E, Casey L, Walker DG, Shum DH. Evaluation of the making sense of brain tumor program: a randomized controlled trial of a home-based psychosocial intervention. Psychooncology. 2015;24(5):540-7.

3. Philip J, Collins A, Panozzo S, Staker J, Murphy M. Mapping the nature of distress raised by patients with high-grade glioma and their family caregivers: a descriptive longitudinal study. Neurooncol Pract. 2020;7(1):103-10.

4. Piderman KM, Breitkopf CR, Jenkins SM, Euerle TT, Lovejoy LA, Kwete GM, et al. A Chaplain-led Spiritual Life Review Pilot Study for Patients with Brain Cancers and Other Degenerative Neurologic Diseases. Rambam Maimonides Med J. 2015;6(2):e0015.

5. Piderman KM, Radecki Breitkopf C, Jenkins SM, Lapid MI, Kwete GM, Sytsma TT, et al. The impact of a spiritual legacy intervention in patients with brain cancers and other neurologic illnesses and their support persons. Psycho-Oncology. 2017;26(3):346-53.

6. Randazzo DM, McSherry F, Herndon JE, 2nd, Affronti ML, Lipp ES, Miller ES, et al. Spiritual well-being and its association with health-related quality of life in primary brain tumor patients. Neurooncol Pract. 2021;8(3):299-309.

NOTE: Some scales (including various modified and translated versions) listed within Appendix 4 have been identified as 'contaminated scales' and should not be utilized or utilized with caution (particularly the FACIT-SP-12). Please refer to Koenig and Carey (2024) for examples of contaminated scales.
